# Morphology, immunohistochemistry characteristics, and clinical presentation of microcystic urothelial carcinoma: a series of 10 cases

**DOI:** 10.1186/s13000-023-01381-1

**Published:** 2023-08-19

**Authors:** Wenjing Su, Wenwen Sui, Xiankui Cheng, Yuanyuan Zong, Yejun Qin, Fengyun Cui

**Affiliations:** 1 Department of Pathology, Shandong Provincial Hospital affiliated to Shandong First Medical University, Jinan, 250021 Shandong P.R. China; 2 Department of Pathology, Dongying District People’s Hospital, Dongying, 257000 Shandong P.R. China

**Keywords:** Urothelial carcinoma, Microcystic variant, Basal, Luminal, Prognosis, *TERT*

## Abstract

**Background:**

Microcystic urothelial carcinoma (MUC) is a rare variant of urothelial carcinoma with histological appearances similar to begin lesions. Thus far, approximately 50 cases have been reported. Here, we investigated the clinicopathological features of MUC.

**Methods:**

Clinical data and paraffin-embedded tissue blocks were collected. Immunohistochemical staining and polymerase chain reaction–Sanger sequencing were performed to detect the phenotype and *TERT* mutation status of MUC, respectively.

**Results:**

The mean patient age was 58.8 ± 14.5 years, with a male predominance (8:2). The pathological stage was T1 in one case, T2 in three cases, T3 in four cases, and T4 in two cases. Tumor metastases or death occurred in all five patients who were followed up within 1–3 years. Histological analyses revealed microcystic, tubular, cribriform, and occasionally cord-like structures, which generally lacked interstitial reactions. The lumens were empty, contained eosinophilic secretion, or were filled with mucin. The microcysts/tubules/cribriform patterns were lined by flat, cuboid, signet ring, or columnar types of epithelia. The cuboid, signet ring, and columnar types represented “glandular metaplasia” or glandular differentiation of urothelial carcinoma. Immunohistochemistry analyses revealed distinct co-expression patterns involving the luminal markers FOXA1 and GATA3, as well as the basal markers CK5/6 and CD44. All 10 cases exhibited a luminal phenotype according to the GATA3+/CK14- criterion, whereas nine cases exhibited a luminal phenotype according to the FOXA1+/CK14- criterion. The telomerase reverse transcriptase-C228T mutation was detected in seven cases.

**Conclusions:**

MUC is a rare variant with a deceptively benign form of urothelial carcinoma, which is generally identified as a late-stage tumor with a poor prognosis. It exhibits distinct co-expression of luminal and basal markers, along with the *TERT*-C228T mutation.

## Background

Microcystic urothelial carcinoma (MUC), a rare variant of urothelial carcinoma (UC), is recognized in the World Health Organization classification of urothelial neoplasms [1] and displays a deceptively benign appearance resembling glandular cystitis. MUC poses an important differential diagnostic challenge, particularly in limited biopsy specimens.

Thus far, limited data are available regarding the clinicopathological features of this pathological entity. Approximately 50 MUC cases have been reported in the English literature, mostly through case reports [2, 3]. To our knowledge, there have been only three major series of reports concerning MUC. The first four MUC cases were described in 1991 [4], along with a comprehensive description of MUC morphology. In 1997, Paz et al. described 12 cases of UC with microcysts; they noted that the pathological stage of most cases was pTa or pT1 [5]. In 2014, Antonio et al. described 20 cases of MUC, along with systematic explanations of their morphology, immunohistochemistry (IHC) characteristics, and clinical presentation [6]. However, minimal data have been available regarding the immunophenotype of MUC. Notably, an analysis of IHC characteristics suggested that MUC was a basal-like UC [7]. Various clinical studies have revealed distinct clinical courses and prognoses of MUC [4–6]. These results presumably reflect the heterogeneity of the reported case series and the incomplete characterization of this rare subtype of UC; thus, additional studies are urgently needed.

Here, we present a series of 10 MUC cases, which is among the largest studies of MUC published thus far. We provide a detailed morphological description of the respective structural patterns. Furthermore, we introduce an immunohistochemical marker panel for characterization of basal and luminal differentiation, and we evaluate the markers in accordance with methods reported in previous studies. Finally, we describe findings concerning the status of telomerase reverse transcriptase (*TERT*) promoter mutations in MUC.

## Methods

Ten cases of MUC were identified among archived formalin-fixed and paraffin-embedded tissue blocks from patients with UC, glandular differentiation, and MUC who received treatment between January 2010 and May 2020 in our institution. Histological details such as epithelial morphology, proportion of MUC in muscle invasive UC, and accompanying types of UC were assessed by two of the authors (WJ Su and FY Cui). Tumor cytology grades and stages were assessed using the World Health Organization criteria [1]. Clinical data were obtained from medical records. When available, follow-up information was obtained.

Immunohistochemical staining was performed using a Dako Link 48 Autostainer, in accordance with the manufacturer’s instructions. Primary antibodies against the following proteins were used: p63 (Maixin-Bio, Fuzhou, China), as well as FOXA1, GATA3, CD44, CK5/6, CK14, CK20, and MUC5AC (all from Zsbio, Beijing, China). Periodic acid Schiff staining after diastasis pre-digestion (PASD) was performed on paraffin sections, in accordance with routine laboratory procedures. IHC interpretations were performed using a combined intensity and percentage scale [8] established by groups who assessed IHC profiles in nested UC [9]. Cytoplasmic staining was scored for CK5/6 and CK20, membrane staining was scored for CD44, and nuclear staining was scored for GATA3 and FOXA1. These markers received a score from 0 to 4 for the percentage of positive tumor cells (0, 0%; 1, < 10%; 2, 10–50%; 3, 51–80%; and 4, > 80%) and a score from 0 to 3 for staining intensity (0, none; 1, slight; 2, medium; and 3, strong). The values were multiplied for a maximum score of 12. An IHC score of > 4 was regarded as a positive result for all markers in the composite assessment because this score was within the range used in previous IHC surrogate studies of bladder cancer [10–12].

*TERT* promoter mutations (C228T and C250T) were detected by polymerase chain reaction and Sanger sequencing. Total DNA was extracted from 10 sections of 5-µm-thick formalin-fixed and paraffin-embedded tissues that contained > 70% of the tumor components. Polymerase chain reaction–Sanger sequencing was performed using the ADx Sanger sequencing platform (AmoyDx, Xiamen, China).

## Results

The clinical features of the 10 cases are shown in Table 1. The mean patient age was 58.8 ± 14.5 years (range: 35–86 years), with a male predominance (8:2). Hematuria, urinary tract infection, lumbago, calculi, and hydronephrosis were noted in eight, nine, two, two, and four cases, respectively. The tumors occurred in the bladder in eight cases and renal pelvis in two cases. The mean tumor diameter was 4.4 ± 2.6 cm (range: 2.5–9.5 cm). Nearly all cases involved extended resection or radical surgery; one case involved simple tumor resection alone. The cases were predominantly (9/10, 90%) muscle-invasive urothelial carcinoma (MIUC). The pathological stages were T1 in one case, T2 in three cases, T3 in four cases, T4 in two cases, and N1 in one case. Of the five patients available for follow-up, three died of the disease and two developed distant metastases within 1–3 years.

**Table 1:** Clinical findings of the 10 MUC cases

Case NO.	Age/sex	Symptoms	Location	Tumor Diameter (cm)	TNM Stage	Operation	Follow Up
1	62y/male	Hematuria, UTI	Bladder	3	T1N0M0	RBPS	LFP
2	71y/male	Hematuria, UTI, lumbago	Bladder	4.5	T4aN0M0	Cystectomy	LFP
3	44y/male	UTI	Bladder	9.5	T3bN0M0	RBPS	LFP
4	58y/male	Hematuria, UTI	Bladder	3	T2aN0M0	Cystectomy	Died/3y
5	47y/male	UTI, hydronephrosis	Bladder	2.5	T2aN0M0	Partial cystectomy	LFP
6	61y/male	Hematuria	Renal pelvis	4	T3N0M0	NUPC	Died/2y
7	57y/female	Hematuria, UTI, lumbago, fever	Renal pelvis	9	T3N0M0	NUPC	LFP
8	67y/male	Hematuria, UTI, calculi, hydronephrosis	Bladder	3	T2bN0M0	Partial cystectomy	Died/1.5y
9	86y/male	Hematuria, UTI, hydronephrosis	Bladder	2.5	T4N0M0	Simple tumor removal	Bone metastasis/1y
10	35y/female	Hematuria, UTI, calculi, hydronephrosis	Bladder	5.5	T3bN1M0	NUPC	Bone, lung and ovary metastasis /2y

UTI: urinary tract infection, RBPS: Resection of bladder, prostate and seminal vesicle, NUPC: nephroureterectomy + partial cystectomy, LFP: lost follow up

The pathological findings of the 10 cases are described in Table 2. Cytology revealed low to intermediate nuclear grade epithelial findings in all cases. Other MIUC components, such as UC with squamous differentiation (1/10, 10%) and nested UC (1/10, 10%) were also present among cases in this study. The proportion of MUC in MIUC ranged from approximately 20–100%. Urothelial dysplasia in the superficial epithelium was detected in all cases.

**Table 2:** Pathological findings of the 10 MUC cases

Case NO.	Structure form	Tumor cytology	Outer/inner epithelium	Urothelial dysplasia	Concomitant MIUC types	PMI
1	Cribriform, microcystic	low-intermediate	Flat/cubic	Yes	-	100%
2	Cribriform, tubular, cord	low	Flat/cubic	Yes	-	100%
3	Microcystic, tubular, cord	low	Flat/cubic	Yes	NUC	20%
4	Tubular, cribriform	low	Flat/cubic, columnar	Yes	-	100
5	Tubular, microcystic, cribriform	low	Flat/cubic, signet ring cell	Yes	-	100%
6	Tubular, cribriform	low-intermediate	Flat/cubic	Yes	-	100%
7	Tubular, microcystic, cribriform	low	Flat/cubic	Yes	-	100%
8	Cord, tubular, cribriform	low-intermediate	Flat/cubic, columnar, mucous epithelium	Yes	squamous differentiation	30%
9	Tubular, microcystic, cord	low	Flat/cubic	Yes	-	100%
10	Tubular, cribriform, cord	low	Flat/cubic	Yes	-	100%

Abbreviations: UC, urothelial carcinoma; CIS, carcinoma in situ; NUC, nested urothelial carcinoma; MIUC, muscle invasive urothelial carcinoma; PMI, proportion of MUC in MIUC

**Fig. 1:**
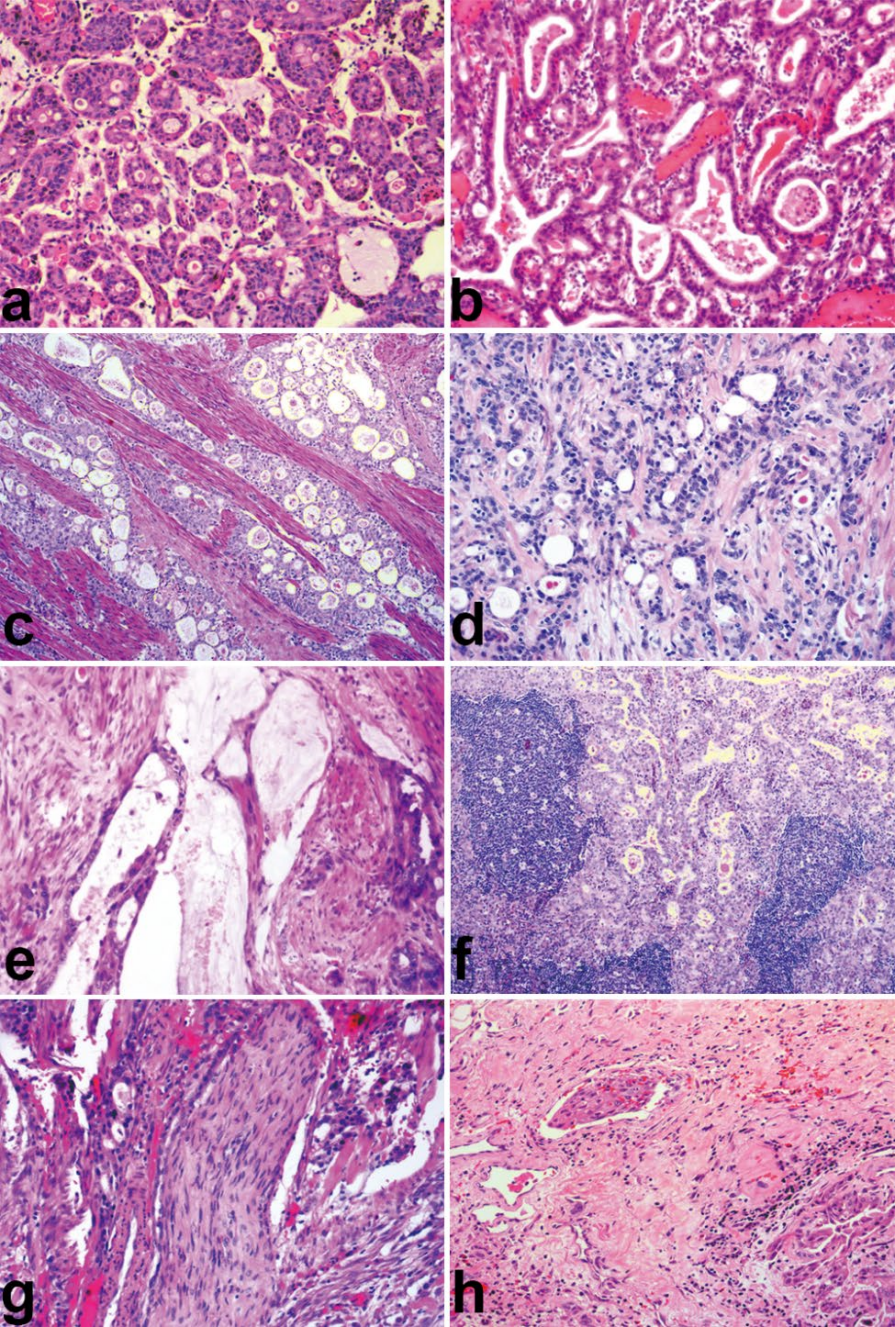
Structural pattern of microcystic urothelial carcinoma. MUC exhibited microcystic (**a**), gland-like tubular (**b**), cribriform (**c**), and occasional cord-like structures (**d**); most areas lacked a stromal response. The cysts and tubules contained eosinophilic secretions (**b**), were empty (**d**), or were filled with mucin (**e**). Lymph node metastases (**f**), perineural invasion (g), and vascular invasion (h) were detected in individual cases

Histological analyses revealed a cystitis glandularis-like pattern at low magnification, which involved characteristic microcysts (Fig. 1a). As shown in Fig. 1(a–e), MUC showed microcystic, gland-like tubular, cribriform, and occasionally cord-like structures; most areas lacked a stromal response. The lumens contained lightly to intensely eosinophilic secretions (Fig. 1b), were empty (Fig. 1d), or filled with mucin (Fig. 1e). Lymph node metastases (Fig. 1f), perineural invasion (Fig. 1g), and vascular invasion (Fig. 1h) were detected in a few cases.

**Fig. 2:**
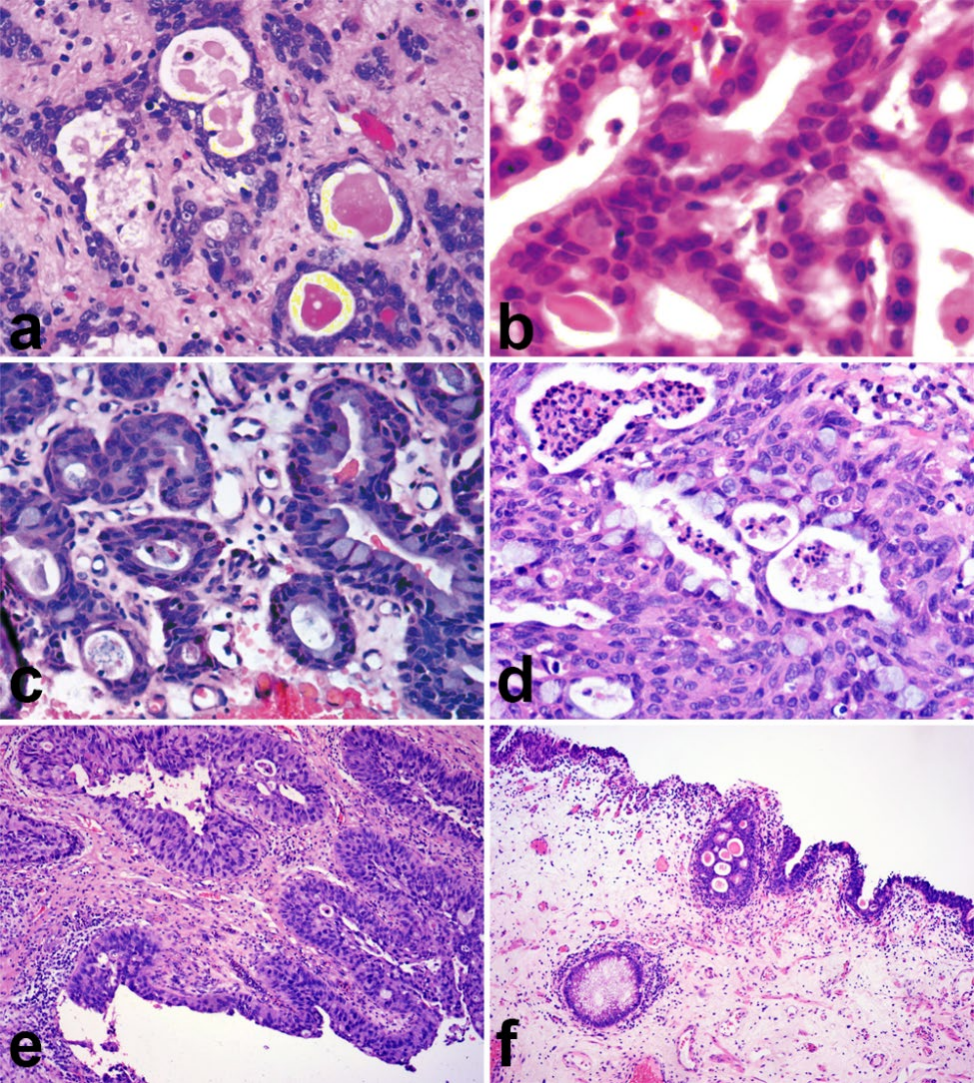
Morphological features of microcystic urothelial carcinoma. The cyst lining comprised epithelium of low to intermediate nuclear grade. Most cysts were lined by one or more layers of cells, which generally exhibited features indicative of transitional cells (**a**). Occasionally, the microcysts were lined by cuboidal cells (**b**), columnar mucinous epithelium (**c**), or signet ring cells (**d**). Urothelial dysplasia (**e**) and glandular urethritis with focal intestinal metaplasia were also detected in the peripheral mucosa (**f**)

Most cysts were lined by one or more layers of cells, which generally exhibited features indicative of transitional cells; the cells lining the cysts showed enlarged nucleoli and coarse nuclear chromatin (Fig. 2a). However, the lumens were often lined by cuboidal cells that contained more abundant eosinophilic cytoplasm (Fig. 2b). Occasionally, the cysts were lined by mucinous columnar epithelium (Fig. 2c) or signet ring cells (Fig. 2d). In all cases, the lining comprised epithelium of low to intermediate nuclear grade. Urothelial dysplasia was observed in the urothelial epithelium adjacent to the carcinoma (Fig. 2e). Glandular urethritis was presented in seven of 10 cases (70%), and the focus of intestinal metaplasia was detected in the peripheral mucosa (Fig. 2f).

**Fig. 3:**
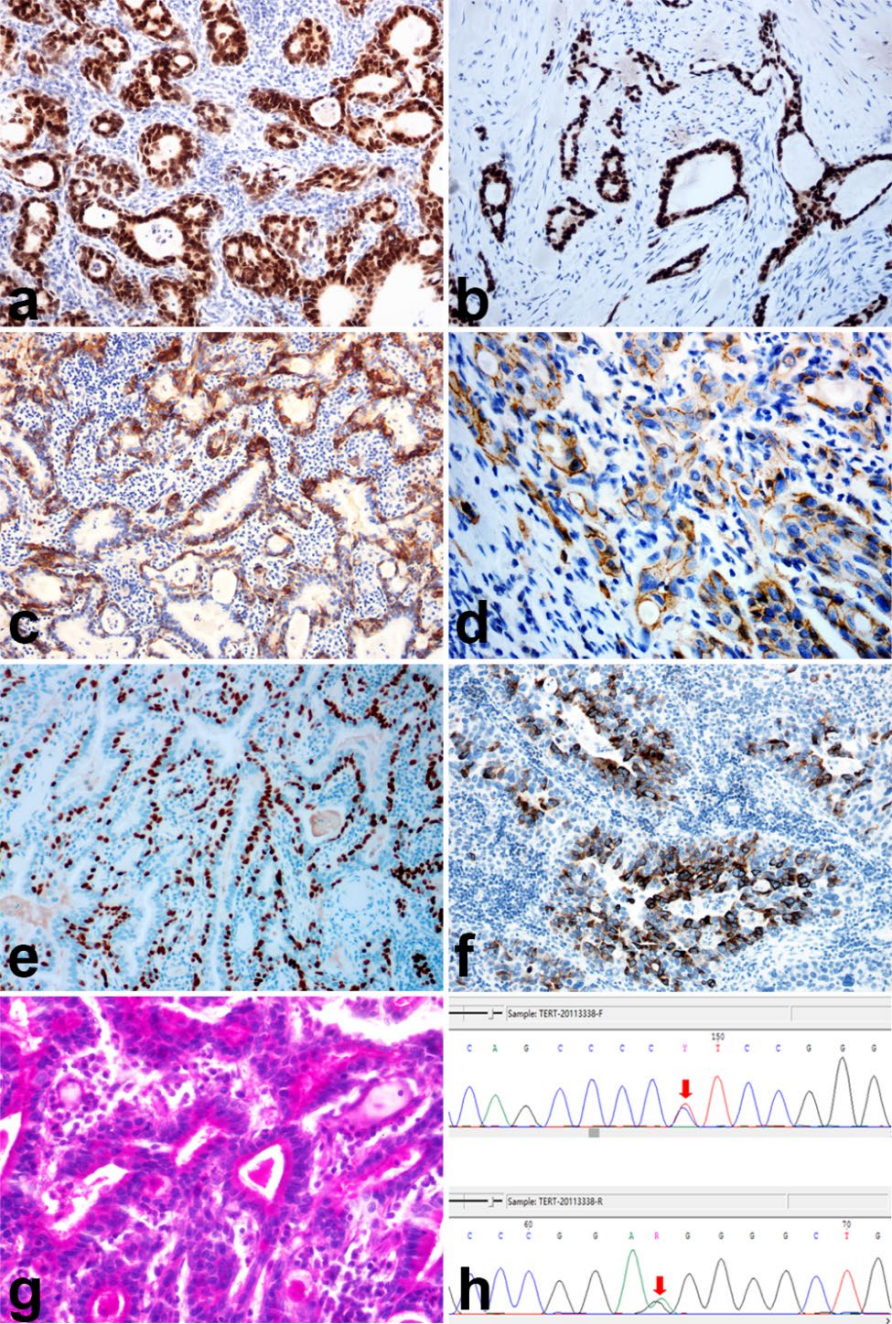
Immunohistochemical features of microcystic urothelial carcinoma. The tumor cells exhibited expression of GATA3 (**a**), FOXA1 (**b**), CK5/6 (**c**, basal location), CD44 (**d**, basal location), p63 (**e**, basal location), and MUC5AC (f, luminal location). PASD staining revealed mucus in the microcysts and cytoplasm (**g**). In most cases, *TERT*-C228T promoter mutations were detected using the ADx Sanger sequencing platform (**h**)

To characterize the phenotype of MUC, the expression patterns of the luminal markers GATA3, FOXA1, and CK20, as well as the basal markers CD44, CK5/6, and CK14, were detected by IHC. The IHC staining scores are shown in Table 3. Positive staining results were observed for the luminal markers GATA3 (10/10, Fig. 3a) and FOXA1 (9/10, Fig. 3b), as well as the basal markers CK5/6 (9/10, Fig. 3c, mainly basal location) and CD44 (5/10, Fig. 3d, mainly basal location). The positive rates of CK20(1/10) and CK14 (0/10) were relatively low in the microcystic components. Additionally, p63, which is widely expressed in most urothelial tumors, showed strong positive staining results in basal and parabasal cells, whereas the staining results were negative for inner gland lumens (Fig. 3e). MUC5AC staining was robust in the lumens of microcystic components, implying glandular differentiation of UC (Fig. 3f). PASD revealed mucus in the microcysts and cytoplasm (Fig. 3g).

**Table 3:** Immunohistochemical staining scores of a series of luminal/basal markers in 10 MUC cases Note: An IHC score greater than 4 was defined as positive for all markers

Case NO.	Luminal markers				Basal markers		
	GATA3	FOXA1	CK20		CD44	CK5/6	CK14
1	6	12	0		2	4	0
2	6	6	0		2	9	4
3	8	12	4		6	9	0
4	9	9	10		2	6	1
5	12	12	0		6	6	2
6	9	9	0		6	9	0
7	6	6	0		3	9	0
8	9	4	2		4	6	2
9	6	6	0		6	9	4
10	6	9	0		6	9	2

The results of classification using IHC-based composite surrogates for molecular tumor subtypes are presented in Table 4. Using the GATA3+/CK5/6- criterion established by Dadhania et al., 1/10 (10%) MUC cases were classified as luminal [12]. Using a GATA3+/CK14- criterion or a FOXA1+/CK14- criterion [9, 12], 10/10 (100%) or 9/10 (90%) cases were classified as luminal. Similarly, using FOXA1 and CK5/6 as a two-marker panel, similar to the method established by Rebouissou et al. [10], 1/10 (10%) cases were classified as non-basal-like (FOXA1+/CK5/6-); 1/10 (10%) cases exhibited a basal phenotype (FOXA1-/CK5/6+). The well-established basal/squamous cell carcinoma-like composite CK5/6+/CK14+/FOXA1-/GATA3- phenotype was not observed in any case [11].

**Table 4:** Composite Immunophenotypic Surrogates for Molecular Subtype in MUC

	Composite Immunophenotype		Classification	Total (n = 10), No. (%)
	Positive (IHC Score > 4)	Negative (IHC Score ≤ 4)		
Dadhania et al. [12]	GATA3	CK5/6	Luminal	1(10)
	CK5/6	GATA3	Basal	0(0)
	GATA3	CK14	Luminal	10(100)
	CK14	GATA3	Basal	0 (0)
Rebouissou et al. [10]	FOXA1	CK5/6	Non–basal-like	1(10)
	CK5/6	FOXA1	Basal-like	1(10)
Sjödahl et al. [11]	CK5/6 and CK14	FOXA1 and GATA3	Basal/SCC-like	0(0)
Johnson et al. [9]	FOXA1	CK14	Luminal	9(90)

The mutation status of the *TERT* promoter in MUC was also detected. The *TERT*-C228T mutation was detected in 7/10 (70%) cases (Fig. 3h). No *TERT*-C250T mutation was detected in any case.

## Discussion

UC exhibits diverse histological patterns. Rare variants of UC (e.g., microcystic, tubular, nested, plasmacytoid, micropapillary, and villoglandular) are included in the World Health Organization classification system [1] because of their unique histological appearance. These variants are important from diagnostic, prognostic, and/or therapeutic perspectives.

MUC is a rare variant of invasive UC with a deceptively benign appearance that features prominent microcysts, macrocysts, or tubular structures. There have been limited reports concerning the clinical presentation and histopathological features of MUC. To our knowledge, the present study is among the most extensive analyses of the structural patterns and cellular features of this unusual form of UC.

In terms of morphology, the MUC components in this study exhibited prominent tubular/microcystic/cribriform structures, unremarkable cell atypia, and the absence of an interstitial reaction; these observations are generally consistent with the findings in previous reports. Nearly all our cases exhibited lumens composed of simple cuboidal epithelium. The cuboidal cells had more abundant eosinophilic cytoplasm, compared with typical neoplastic urothelial cells. Robert et al. described a similar phenomenon, which they named transitional cell carcinoma with “glandular areas” or “glandular metaplasia” [4]. Furthermore, we identified mucous columnar cells lining the microcysts, which we regarded as definite glandular differentiation of UC. Robert et al. [4] also encountered this morphology, in which cells that lined the lumens were low columnar epithelium; the cells contained intracytoplasmic mucin in three of the four cases. and in one of our 10 cases, signet ring cells with microcytic lumens were present, which diverged from the typical features of microcystic UC. Isabel et al. reported a multicystic UC of the bladder with signet-ring cells [2], and Yuji et al. described a UC of the bladder that contained 20% signet ring cells [13]. We presumed that the signet ring cell component comprised another type of glandular differentiation of UC. Notably, Antonio et al. mentioned that some cysts were lined by one or more layers of low columnar cells; the expression of MUC5AC, a marker of glandular differentiation in UC, was mainly observed around lumens [6]. Considering these past and present findings, “glandular metaplasia” or “glandular differentiation” may be more frequent in MUC than in conventional UC. UC with glandular differentiation tends to be more biologically aggressive and have a greater tendency to produce extravesical tumors and node-positive disease. However, there is evidence that glandular differentiation in UC does not influence prognosis after adjustment for staging [14, 15].

The need to predict patient outcomes has driven molecular studies in both invasive and noninvasive UC. Large-scale transcriptomic studies of MIUC suggest that most MIUC cases can be divided into two groups: luminal and basal [16, 17]. These groups can be used to predict prognosis [16, 18] and potential response to chemotherapy [19]. The use of immunohistochemical surrogates to identify the molecular subtypes of MIUC has been validated by several groups. These studies have shown that reliable discrimination among molecular phenotypes of conventional UC can be achieved by using various basal and luminal markers [11, 12]. Generally, CK5/6, CK14, and CD44 have been used as basal markers, whereas FOXA1, GATA3, and CK20 have been used as luminal markers [16, 20].

Thus far, there have been few reports concerning the immunophenotype of MUC, particularly in terms of immunohistochemical surrogates for MUC molecular subtypes. One group reported double-positive staining of the basal markers CK5 and CD44 in all 14 cases of a combined cohort of small nested and microcystic variants of MIUC [7]. However, the specific numbers of cases and the staining patterns of MUC were not reported in that study; moreover, simultaneous detection of luminal markers was not performed.

In the present study, we analyzed cases of MUC using a small series of IHC markers that were previously validated for basal and luminal subtypes of MIUC. We found distinct co-expression of the luminal markers FOXA1 and GATA3 in MUC, as well as the basal markers CK5/6 and CD44. Nine of the 10 cases exhibited a basally oriented CK5/6 staining pattern, which has previously been identified as a feature of CK5 expression in MIUC with an “urothelial-like” gene expression profile [11, 21]; those cases also retained diffuse expression of the luminal markers FOXA1 and GATA3. The overall immunophenotypic profile suggests that MUC harbors a urothelial-like molecular signature. An identical FOXA1/GATA3/CK5/6 staining pattern was identified in a cohort of nested UC cases [9, 21, 22]; this pattern may represent a distinct subtype within the larger luminal classification schema. However, MUC can be converted to a luminal or basal subtype, as indicated by the 2017 The Cancer Genome Atlas molecular subtype; it can also be converted to a luminal unstable or basal/squamous subtype, as described by the European Association of Urology and European Society for Medical Oncology consensus [23]. Thus, our results highlight the challenges of using IHC surrogates for analyses of molecular phenotype. Larger cohorts and dedicated gene expression profiles in MUC are warranted to determine the feasibility of routine clinical use of IHC classifiers; such data may provide further insights regarding the mechanisms that underlie this unique variant.

*TERT* promoter mutations are among the most frequently mutated genomic regions in many types of malignant tumors such as glioblastoma, hepatocellular carcinoma, thyroid carcinoma, and UC [24]. The respective hotspot mutations C228T and C250T are located 124 and 146 bp upstream of the translation start site of the *TERT* gene. In UC, the *TERT* promoter is among the most frequently mutated genomic regions, along with FGFR3 and TP53 [25]. *TERT* promoter mutations are often detected in precancerous UC lesions and high grade/stage urothelial bladder cancer, or in rare variant pathologies with an aggressive phenotype. The respective frequencies of *TERT* promoter mutations in UC, adenocarcinoma, micropapillary variants, and plasmacytoid variants are 52%, 57%, 100%, and 60%, although such mutations are usually absent from potentially benign mimics (e.g., von Brunn nests, cystitis cystica, and nephrogenic adenoma) and metastatic carcinoma [26, 27]. Thus, the present findings further support the utility of *TERT* promoter mutation analysis for differential diagnosis, particularly when limited biopsy tissue is available.

To our knowledge, the *TERT* promoter mutation status in MUC has been rarely reported [28]. In the present study, we found that MUC had a high frequency (7/10) of *TERT*-C228T mutations, but there were no C250T mutations. Although the detailed mechanism underlying the biological effects of the C228T mutation remains unclear, there is evidence that the C228T mutation produces a more aggressive malignant phenotype, compared with the C250T mutation. Borah et al. showed that mutations in the *TERT* promoter, mainly involving C228T, were associated with high levels of *TERT* mRNA expression, *TERT* protein expression, telomerase activity, and telomere length in bladder cancer cell lines [29]. Additionally, the presence of the C228T mutation in urinary cell-free DNA was associated with bladder tumor recurrence in patients who had undergone transurethral surgery for non-muscle-invasive bladder cancer or radical nephroureterectomy for localized upper tract UC [30, 31].

Biological analyses suggest that, after stage matching, the prognosis for the microcystic variant is similar to the prognosis for conventional UC [6]. Lopez et al. described 20 patients with MUC who underwent surgery; 11 of these patients died of the disease after a mean interval of 30 months (range: 11–56 months), yielding a mortality rate of > 50% [6]. In the present study, tumor metastasis or death occurred within 1–3 years in all five patients for whom follow-up data were available. However, because of the small number of patients and short follow-up interval, no definitive conclusions could be made regarding the prognostic value of the microcystic variant or the proportion of microcystic components throughout each tumor; larger studies are needed to determine the appropriate course of treatment. Additional cases are needed to determine the effects of tumor location, proportion of MUC throughout each tumor, and pathological stages on prognosis.

## Conclusions

In summary, MUC is a variant of UC with diagnostic, prognostic, and therapeutic significance. The probability of glandular differentiation appears to be much higher in MUC than in conventional UC. MUC exhibits distinct co-expression of the luminal markers FOXA1 and GATA3, as well as the basal markers CK5/6 and CD44. Further studies are needed to define the prognostic significance and therapeutic value of MUC.

## Data Availability

All data generated or analyzed during this study are included in this published article.

## Abbreviations

MUC: Microcystic urothelial carcinoma
UC: Urothelial carcinoma
IHC: Immunohistochemistry
*TERT*: Telomerase reverse transcriptase
PASD: Periodic acid Schiff staining after diastasis pre-digestion
MIUC: Muscle-invasive urothelial carcinoma

## References

[1] Moch H, Humphrey PA, Ulbright TM (2016). WHO classification of Tumours of the urinary system and male genital Organs.

[2] Alvarado-Cabrero I, Pérez-Montiel D, Hes O (2008). Multicystic urothelial carcinoma of the bladder with gland-like lumina and with signet-ring cells. A case report. Diagn Pathol.

[3] Kadouri Y, Zaoui Y, Derkaoui S, El Sayegh H, Benslimane L, Nouini Y (2020). Microcystic urothelial carcinoma of the bladder: a case report. Urol Case Rep.

[4] Young RH, Zukerberg LR (1991). Microcystic transitional cell carcinomas of the urinary bladder. A report of four cases. Am J Clin Pathol.

[5] Paz A, Rath-Wolfson L, Lask D, Koren R, Manes A, Mukamel E (1997). The clinical and histological features of transitional cell carcinoma of the bladder with microcysts: analysis of 12 cases. Br J Urol.

[6] Lopez Beltran A, Montironi R, Cheng L (2014). Microcystic urothelial carcinoma: morphology, immunohistochemistry and clinical behaviour. Histopathology.

[7] Mai KT, Hakim SW, Ball CG, Flood TA, Belanger EC (2014). Nested and microcystic variants of urothelial carcinoma displaying immunohistochemical features of basal-like urothelial cells: an immunohistochemical and histopathogenetic study. Pathol Int.

[8] Remmele W, Stegner HE (1987). [Recommendation for uniform definition of an immunoreactive score (IRS) for immunohistochemical estrogen receptor detection (ER-ICA) in breast cancer tissue]. Pathologe.

[9] Johnson SM, Khararjian A, Legesse TB, Khani F, Robinson BD, Epstein JI (2021). Nested variant of Urothelial Carcinoma is a luminal bladder tumor with distinct coexpression of the basal marker cytokeratin 5/6. Am J Clin Pathol.

[10] Rebouissou S, Bernard-Pierrot I, de Reyniès A, Lepage ML, Krucker C, Chapeaublanc E (2014). EGFR as a potential therapeutic target for a subset of muscle-invasive bladder cancers presenting a basal-like phenotype. Sci Transl Med.

[11] Sjödahl G, Eriksson P, Liedberg F, Höglund M (2017). Molecular classification of urothelial carcinoma: global mRNA classification versus tumour-cell phenotype classification. J Pathol.

[12] Dadhania V, Zhang M, Zhang L, Bondaruk J, Majewski T, Siefker-Radtke A (2016). Meta-analysis of the luminal and basal subtypes of bladder Cancer and the identification of signature immunohistochemical markers for clinical use. EBioMedicine.

[13] Ohtsuki Y, Fukumoto T, Okada Y, Teratani Y, Hayashi Y, Lee GH (2010). Immunohistochemical and ultrastructural characterization of the signet-ring cell carcinoma component in a case of urothelial carcinoma of the urinary bladder. Med Mol Morphol.

[14] Wasco MJ, Daignault S, Zhang Y, Kunju LP, Kinnaman M, Braun T (2007). Urothelial carcinoma with divergent histologic differentiation (mixed histologic features) predicts the presence of locally advanced bladder cancer when detected at transurethral resection. Urology.

[15] Kim SP, Frank I, Cheville JC, Thompson RH, Weight CJ, Thapa P (2012). The impact of squamous and glandular differentiation on survival after radical cystectomy for urothelial carcinoma. J Urol.

[16] Choi W, Porten S, Kim S, Willis D, Plimack ER, Hoffman-Censits J (2014). Identification of distinct basal and luminal subtypes of muscle-invasive bladder cancer with different sensitivities to frontline chemotherapy. Cancer Cell.

[17] McConkey DJ, Choi W (2018). Molecular subtypes of bladder Cancer. Curr Oncol Rep.

[18] Damrauer JS, Hoadley KA, Chism DD, Fan C, Tiganelli CJ, Wobker SE (2014). Intrinsic subtypes of high-grade bladder cancer reflect the hallmarks of breast cancer biology. Proc Natl Acad Sci U S A.

[19] Seiler R, Ashab H, Erho N, van Rhijn B, Winters B, Douglas J (2017). Impact of Molecular Subtypes in muscle-invasive bladder Cancer on Predicting Response and Survival after Neoadjuvant Chemotherapy. Eur Urol.

[20] Lerner SP, McConkey DJ, Hoadley KA, Chan KS, Kim WY, Radvanyi F (2016). Bladder Cancer Molecular Taxonomy: Summary from a Consensus Meeting. Bladder Cancer.

[21] Warrick JI, Kaag M, Raman JD, Chan W, Tran T, Kunchala S (2017). FOXA1 and CK14 as markers of luminal and basal subtypes in histologic variants of bladder cancer and their associated conventional urothelial carcinoma. Virchows Arch.

[22] Kamoun A, de Reyniès A, Allory Y, Sjödahl G, Robertson AG, Seiler R (2020). A Consensus Molecular classification of muscle-invasive bladder Cancer. Eur Urol.

[23] Takahara T, Murase Y, Tsuzuki T (2021). Urothelial carcinoma: variant histology, molecular subtyping, and immunophenotyping significant for treatment outcomes. Pathology.

[24] Killela PJ, Reitman ZJ, Jiao Y, Bettegowda C, Agrawal N, Diaz LA (2013). TERT promoter mutations occur frequently in gliomas and a subset of tumors derived from cells with low rates of self-renewal. Proc Natl Acad Sci U S A.

[25] Pietzak EJ, Bagrodia A, Cha EK, Drill EN, Iyer G, Isharwal S (2017). Next-generation sequencing of nonmuscle invasive bladder Cancer reveals potential biomarkers and rational therapeutic targets. Eur Urol.

[26] Hayashi Y, Fujita K, Netto GJ, Nonomura N (2021). Clinical application of TERT promoter mutations in Urothelial Carcinoma. Front Oncol.

[27] Taylor AS, McKenney JK, Osunkoya AO, Chan MP, Al-Ahmadie HA, Spratt DE (2020). PAX8 expression and TERT promoter mutations in the nested variant of urothelial carcinoma: a clinicopathologic study with immunohistochemical and molecular correlates. Mod Pathol.

[28] Lopez-Beltran A, Henriques V, Montironi R, Cimadamore A, Raspollini MR, Cheng L (2019). Variants and new entities of bladder cancer. Histopathology.

[29] Borah S, Xi L, Zaug AJ, Powell NM, Dancik GM, Cohen SB (2015). Cancer. TERT promoter mutations and telomerase reactivation in urothelial cancer. Science.

[30] Hayashi Y, Fujita K, Matsuzaki K, Eich ML, Tomiyama E, Matsushita M (2020). Clinical significance of Hotspot Mutation analysis of urinary cell-free DNA in urothelial bladder Cancer. Front Oncol.

[31] Hayashi Y, Fujita K, Matsuzaki K, Matsushita M, Kawamura N, Koh Y (2019). Diagnostic potential of TERT promoter and FGFR3 mutations in urinary cell-free DNA in upper tract urothelial carcinoma. Cancer Sci.

